# Supplementary material for: Modeling glioblastoma heterogeneity as a dynamic network of cell states
Source: Mol Syst Biol. 2021 Sep 16;17(9):e10105. doi: 10.15252/msb.202010105 (PMC8444284; doi:10.15252/msb.202010105)
Supplement: Supplementary file 6 — Source Data for Figure 5 [file MSB-17-e10105-s004.zip › Figure5A_sourcedata/GSEA_3071/nosigdb_stateVI.GseaPreranked.1622795169056/RICHARDS_NEFTEL_CELL_2019_MES2.html]

Details for gene set RICHARDS\_NEFTEL\_CELL\_2019\_MES2[GSEA]

|  || Dataset | state63071 |
| Phenotype | NoPhenotypeAvailable |
| Upregulated in class | na\_pos |
| GeneSet | RICHARDS\_NEFTEL\_CELL\_2019\_MES2 |
| Enrichment Score (ES) | 0.4744667 |
| Normalized Enrichment Score (NES) | 2.2185507 |
| Nominal p-value | 0.0 |
| FDR q-value | 0.0030791252 |
| FWER p-Value | 0.01 |
Table: GSEA Results Summary

  

Fig 1: Enrichment plot: RICHARDS\_NEFTEL\_CELL\_2019\_MES2      
 Profile of the Running ES Score & Positions of GeneSet Members on the Rank Ordered List

  

| PROBE | GENE SYMBOL | GENE\_TITLE | RANK IN GENE LIST | RANK METRIC SCORE | RUNNING ES | CORE ENRICHMENT || 1 | DDIT3 |  |  | 14 | 1.086 | 0.0877 | Yes |
| 2 | ANXA2 |  |  | 79 | 0.783 | 0.1361 | Yes |
| 3 | AKAP12 |  |  | 92 | 0.743 | 0.1954 | Yes |
| 4 | PPP1R15A |  |  | 98 | 0.738 | 0.2563 | Yes |
| 5 | SQSTM1 |  |  | 147 | 0.667 | 0.2993 | Yes |
| 6 | LDHA |  |  | 197 | 0.604 | 0.3367 | Yes |
| 7 | SERTAD1 |  |  | 380 | 0.496 | 0.3283 | Yes |
| 8 | TRIB3 |  |  | 387 | 0.495 | 0.3683 | Yes |
| 9 | HSPA5 |  |  | 414 | 0.484 | 0.4020 | Yes |
| 10 | ATF3 |  |  | 416 | 0.483 | 0.4425 | Yes |
| 11 | PFKP |  |  | 473 | 0.463 | 0.4660 | Yes |
| 12 | HERPUD1 |  |  | 830 | 0.371 | 0.3989 | Yes |
| 13 | MT1X |  |  | 867 | 0.366 | 0.4198 | Yes |
| 14 | VIM |  |  | 897 | 0.361 | 0.4423 | Yes |
| 15 | HSPA9 |  |  | 1087 | 0.333 | 0.4181 | Yes |
| 16 | EPAS1 |  |  | 1091 | 0.332 | 0.4452 | Yes |
| 17 | PGK1 |  |  | 1099 | 0.331 | 0.4712 | Yes |
| 18 | ADM |  |  | 1185 | 0.318 | 0.4745 | Yes |
| 19 | SLC2A1 |  |  | 1606 | 0.268 | 0.3810 | No |
| 20 | RPL21 |  |  | 1725 | 0.258 | 0.3701 | No |
| 21 | BNIP3 |  |  | 1778 | 0.253 | 0.3771 | No |
| 22 | IGFBP3 |  |  | 3588 | -1.623 | 0.0138 | No |
Table: GSEA details [plain text format]

  

Fig 2: RICHARDS\_NEFTEL\_CELL\_2019\_MES2: Random ES distribution      
 Gene set null distribution of ES for **RICHARDS\_NEFTEL\_CELL\_2019\_MES2**
